# Synthetic miRNA-Mowers Targeting miR-183-96-182 Cluster or miR-210 Inhibit Growth and Migration and Induce Apoptosis in Bladder Cancer Cells

**DOI:** 10.1371/journal.pone.0052280

**Published:** 2012-12-17

**Authors:** Yuchen Liu, Yonghua Han, Hu Zhang, Liping Nie, Zhimao Jiang, Pingping Fa, Yaoting Gui, Zhiming Cai

**Affiliations:** 1 Guangdong and Shenzhen Key Laboratory of Male Reproductive Medicine and Genetics, Institute of Urology, Peking University Shenzhen Hospital, Shenzhen PKU-HKUST Medical Center, Shenzhen, People's Republic of China; 2 Anhui Medical University, Hefei, People's Republic of China; 3 Shenzhen Key Laboratory of Genitourinary Tumor, Shenzhen Second People's Hospital, First Affiliated Hospital of Shenzhen University, Shenzhen, People's Republic of China; 4 Department of Clinical Laboratory, Peking University Shenzhen Hospital, Shenzhen, People's Republic of China; Queen Elizabeth Hospital, Hong Kong

## Abstract

**Background:**

MicroRNAs (miRNAs) function as endogenous regulators of biological behaviors of human cancers. Several natural non-coding RNAs are reported to inhibit miRNAs by base-pairing interactions. These phenomena raise questions about the ability of artificial device to regulate miRNAs. The purpose of this study is to create synthetic devices that target a single miRNA or a miRNA cluster and to ascertain their therapeutic effects on the phenotypes of bladder cancer cells.

**Methodology/Principal Findings:**

Tandem bulged miRNA binding sites were inserted into the 3′ untranslated region (UTR) of the SV-40 promoter-driven Renilla luciferase gene to construct two “miRNA-mowers” for suppression of miR-183-96-182 cluster or miR-210. A third device with tandem repeat sequences not complementary to any known miRNA was generated as an untargeted-control. In functional analyses, bladder cancer T24 and UM-UC-3 cells were transfected with each of the three devices, followed by assays for detection of their impacts. Luciferase assays indicated that the activities of the luciferase reporters in the miRNA-mowers were decreased to 30–50% of the untargeted-control. Using Real-Time qPCR, the expression levels of the target miRNAs were shown to be reduced 2-3-fold by the corresponding miRNA-mower. Cell growth, apoptosis, and migration were tested by MTT assay, flow cytometry assay, and in vitro scratch assay, respectively. Cell growth inhibition, increased apoptosis, and decreased motility were observed in miRNA-mowers-transfected bladder cancer cells.

**Conclusions/Significance:**

Not only a single target miRNA but also the whole members of a target miRNA cluster can be blocked using this modular design strategy. Anti-cancer effects are induced by the synthetic miRNA-mowers in the bladder cancer cell lines. miR-183/96/182 cluster and miR-210 are shown to play oncogenic roles in bladder cancer. A potentially useful synthetic biology platform for miRNA loss-of-function study and cancer treatment has been established in this work.

## Introduction

Transitional cell carcinoma of the bladder is the most common urinary tract cancer in Eastern and Western countries [1]. The majority of bladder cancers are low-grade non-invasive tumors which may progress to the invasive phenotype. In contrast to non-invasive bladder cancers, muscle-invasive tumors tend to metastasize to other organs and have a very poor prognosis [2]–[3]. The most common treatments for bladder cancer are surgery, chemotherapy, immunotherapy and radiation therapy. However, they are far from satisfactory due to several factors, including lack of effectiveness, absence of specificity, and full of unpleasant side effects [4]–[5]. So there is a growing need for the development of new ways to treat cancer. Several new antineoplastic therapies are currently under experimental and clinical investigation, but no major breakthroughs have been achieved with these therapeutic strategies [6].

It has long been proposed that cancer cells can be re-programmed by assembling different DNA or RNA parts into novel devices to give rise to a benign biological behavior [7]–[8]. Synthetic biology therapy with multiple devices directed at cancer-specific gene pathways opens promising new avenues to improve cancer treatment [9]. On the basis of a detailed understanding of the genetic profiles of cancers, synthetic biologists try to produce predictable and robust biological devices with novel treatment functionalities that do not exist in nature. Although this field is relatively new and is still in the laboratory testing phase, some of the related works have already shown great potentials in the treatments of various types of cancers [10]–[11].

MicroRNAs (miRNAs), a class of short endogenous RNAs, regulate gene expression by binding to partially complementary sequences in the 3′UTR of mRNA [12]. Numerous studies have reported that miRNAs are involved in the development and progression of human cancers, including growth, apoptosis, invasion, and metastasis [13]–[14]. In our previous work we determined the genome-wide miRNA profiles in human bladder cancer by deep sequencing. miR-183-96–182 cluster and miR-210 were found to be up-regulated in human bladder cancer, suggesting that they may play important roles as oncogenes in this cancer [15].

One of the major objectives of our synthetic biology research is to connect synthetic genetic devices to the control of a tumor cell phenotype. In this paper, we present two useful genetic devices–the miR-183-96-182-cluster-mower (miRM-183/96/182) and the miR-210-mower (miRM-210)–that target miRNAs with the partially complementary sequences. We have also investigated their therapeutic effects on the phenotypes of bladder cancer cells. This approach provides a potentially useful synthetic biology platform for miRNA loss-of-function study and cancer treatment.

## Results

### Design and construction of the miRNA-mowers

**Figure 1:**
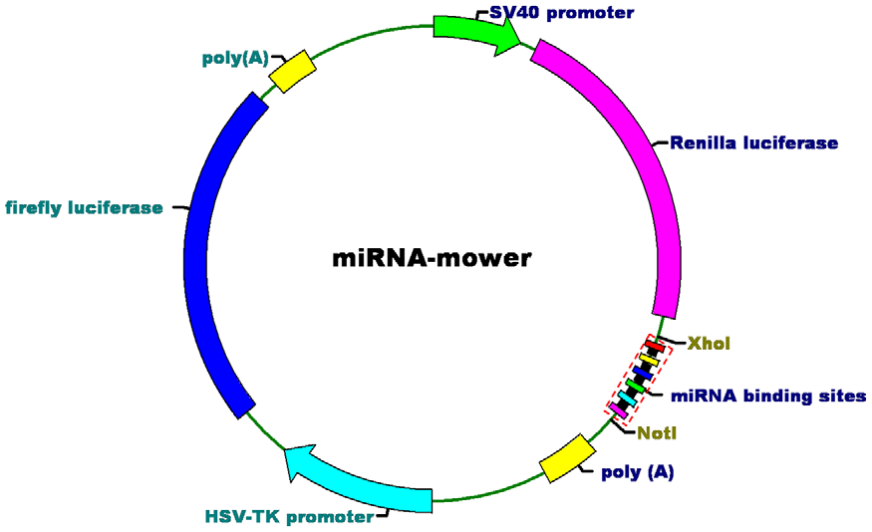
Construction of the miRNA-mowers. We constructed miRNA-mowers by inserting multiple miRNA binding sites into the 3′ UTR of a Renilla luciferase gene. Each construct under control of a SV-40 promoter ended with a SV40 polyadenylation signal. An unregulated TK promoter-driven gene encoding firefly luciferase was used as a control. The black squares represented the linkers between the binding sites.

Inspired by the observations that some RNA molecules expressed from *Herpesvirus saimiri* or the human pseudogenes regulate the endogenous miRNAs by base-pairing interactions in mammalian cells [16]–[18], we have created miRNA-mowers containing binding sites partially complementary to the target miRNAs for miRNA loss-of-function studies in human bladder cancer cells (Fig. 1). To form a more stable interaction with the miRNA, we designed multiple binding sites of miRNA of interest with a central bulge at the cleavage positions of Argonaute 2 [19]. This claim is also based on the discovery that partial pairing between miRNAs and their target sequences is more common in animals than in plants [12]. The binding sites were connected by “linkers” (a few nucleotides) for the purposes of enhancing the binding of miRNA-protein complexes (miRNPs) to every possible binding site, and increasing miRNA knockdown efficiency [20].

We have made 6 tandemly arrayed copies of binding sites for the miR-183-96-182 cluster (2 copies for each miRNA of the cluster). The miRM-183/96/182 was then constructed by inserting these binding sites into the 3′ UTR of a SV-40 promoter-driven luciferase reporter gene in the siCHECKTM-2 vector (Promega, Madison, WI, USA). To demonstrate the modularity of the devices, we inserted another 6 tandemly arrayed copies of binding sites for miR-210 into the 3′ UTR of reporter gene to generate miRM-210 by using the same vector. As an untargeted-control, we also created a device with 6 tandem repeated sequences not complementary to any known miRNAs. The sequences used in this study were available in Table S1 and Table S2.

### Decreased activities of luciferase reporters in miRNA-mowers may contribute to the miRNA binding sites

To test efficacy of the miRNA–mowers, we assayed Renilla luciferase activity relative to firefly luciferase activity in T24 or UM-UC-3 cells 48 h after transfection with each device. The luciferase reporter assays showed that the activities of the reporters containing the bulged miRNA binding sites were decreased in bladder cancer cells, compared with the untargeted-control (P<0.01 for each group). The inhibitions (%) of luciferase expressions were shown in Table 1.

**Table:** **Table1.** The inhibitory effects of endogenous miRNAs on the activities of the luciferase reporters in the miRNA-mowers.

Cell lines	miRNA-mowers	Inhibitions (%)^a^
**T24**	**miRM-183/96/182**	48.7±3.23
**T24**	**miRM-210**	70.0±4.82
**UM-UC-3**	**miRM-183/96/183**	51.4±3.36
**UM-UC-3**	**miRM-210**	73.4±5.12

a The values are mean ± SD. Each experiment in the two cell lines was performed in triplicate for three independent times.

### The miRNA-mowers achieved effective suppression of target miRNA levels in bladder cancer cell lines

To further investigate the functionality of miRNA-mowers, we examined the relative expression levels of the target miRNAs in the devices-transfected human bladder cancer T24 and UM-UC-3 cells. The qPCR results demonstrated that expression of the corresponding miRNA-mower induced a dramatic decrease in expression levels of the target miRNAs in the two bladder cancer cell lines (P<0.05 for each group). The inhibitions (%) of miRNA expressions were shown in Table 2. The expression levels of miR-96, miR-182, and miR-183 could not be suppressed by miRM-210. At the same time, the expression level of miR-210 also could not be changed by miRM-183/96/182. Results of the qPCR experiment were shown in Table S3.

**Table:** **Table2.** The inhibitory effects of miRNA-mowers on the expressions of target miRNAs in bladder cancer cells.

		Inhibitions (%)^a^			
Cell lines	miRNA- mowers	miR-96	miR-182	miR-183	miR-210
**T24**	**miRM- 183/96/182**	49.33±2.46	47.34±4.23	52.67±5.11	−3.42±4.23
**T24**	**miRM-210**	2.42±3.85	−2.13±3.51	3.19±4.25	69.67±3.58
**UM- UC-3**	**miRM- 183/96/182**	46.33±3.57	46.13±3.41	51.37±3.89	2.55±3.52
**UM- UC-3**	**miRM-210**	1.78±3.31	2.33±3.03	−2.39±2.63	70.65±4.24

a The values are mean ± SD. Each experiment in the two cell lines was performed in triplicate for three independent times.

### Inhibition of cell growth induced by the miRNA-mowers in bladder cancer cell lines

**Figure 2:**
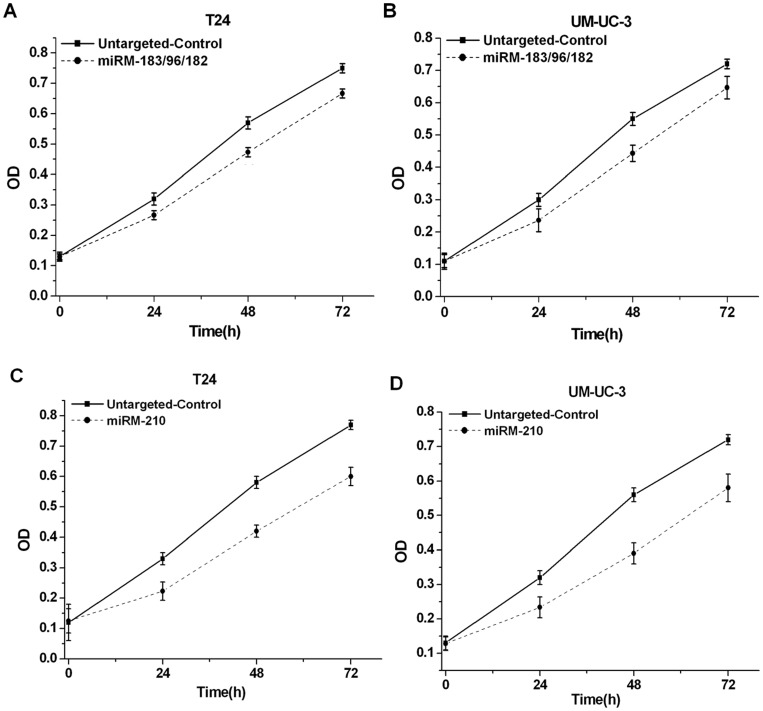
Effects of the synthetic miRNA-mowers on growth of bladder cancer cells. T24 and UM-UC-3 cells were transfected with the devices in 96-well plates. Cell growth was measured by MTT assay at different time intervals. ANOVA was used for the comparison of curves of cell growth. (A) miRM-183/96/182 inhibited cell growth in T24 cells (P<0.05). (B) miRM-183/96/182 inhibited cell growth in UM-UC-3 cells (P<0.05). (C) miRM-210 inhibited cell growth in T24 cells (P<0.01). (D) miRM-210 inhibited cell growth in UM-UC-3 cells (P<0.01). Data were the average of three independent experiments; bars, SD.

Human bladder cancer cell lines T24 and UM-UC-3 were transiently transfected with the devices in 96-well plates. In both bladder cell lines, miRM-183/96/182 or the miRM-210 decreased MTT reactivity (Fig. 2). Such observation could either indicate a cell growth arrest or cell death.

### Initiation of cell apoptosis induced by the miRNA-mowers in bladder cancer cell lines

**Figure 3:**
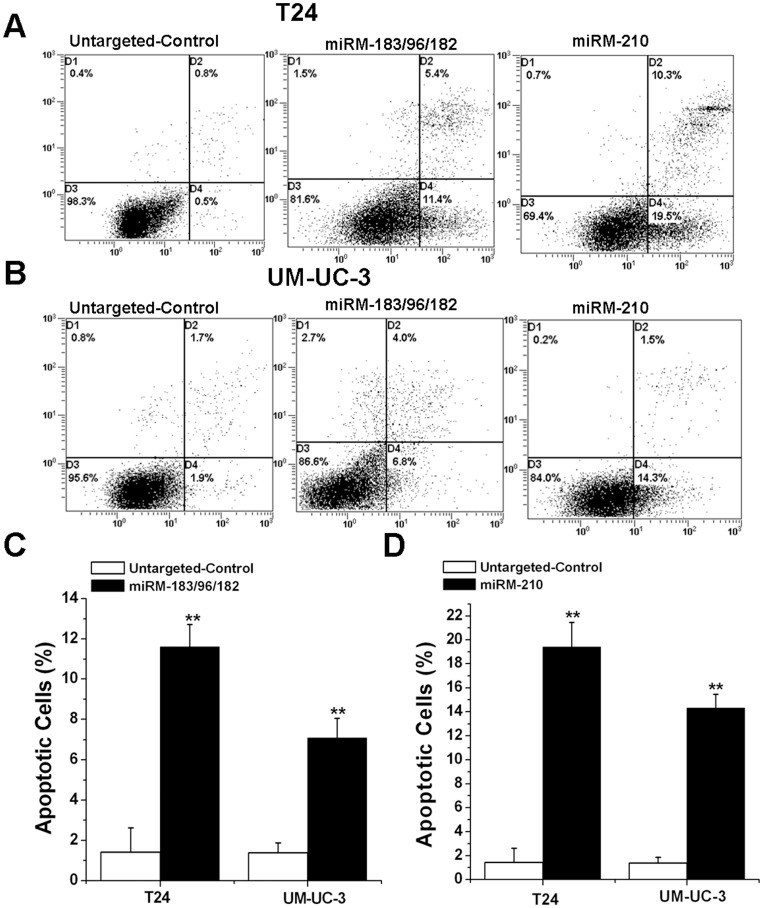
Effects of the synthetic miRNA-mowers on apoptosis of bladder cancer cells. T24 and UM-UC-3 cells were transfected with the devices in 6-well plates. Cell apoptosis was measured by the flow cytometry at 48 h post transfection. (A). Representative images of flow cytometry analysis in T24 cells. (B). Representative images of flow cytometry analysis in UM-UC-3 cells. (C). Cell apoptosis induction was observed in miRM-183/96/182-transfected bladder cancer T24 and UM-UC-3 cells using flow cytometry analysis. (D). Cell apoptosis induction was observed in miRM-210-transfected bladder cancer T24 and UM-UC-3 cells using flow cytometry analysis. We performed each experiment at least three times. Error bars, SD. ** P<0.01, compared with the untargeted-control.

To test cell death, apoptosis experiments were performed. Both of the two cell lines were seeded in six-well plates and transfected with devices. We conducted apoptosis assays using an Annexin V-PE apoptosis detection kit to determine whether the two types of our synthetic miRNA-mowers induce cell apoptosis in bladder cancer cells. The results shown in Fig. 3 demonstrated that the apoptotic cells (%) of T24 and UM-UC-3 cell lines transfected with the miRM-183/96/182 or the miRM-210 were higher than those transfected with the untargeted-control.

### Inhibition of cell migration induced by the miRNA-mowers in bladder cancer cell lines

**Figure 4:**
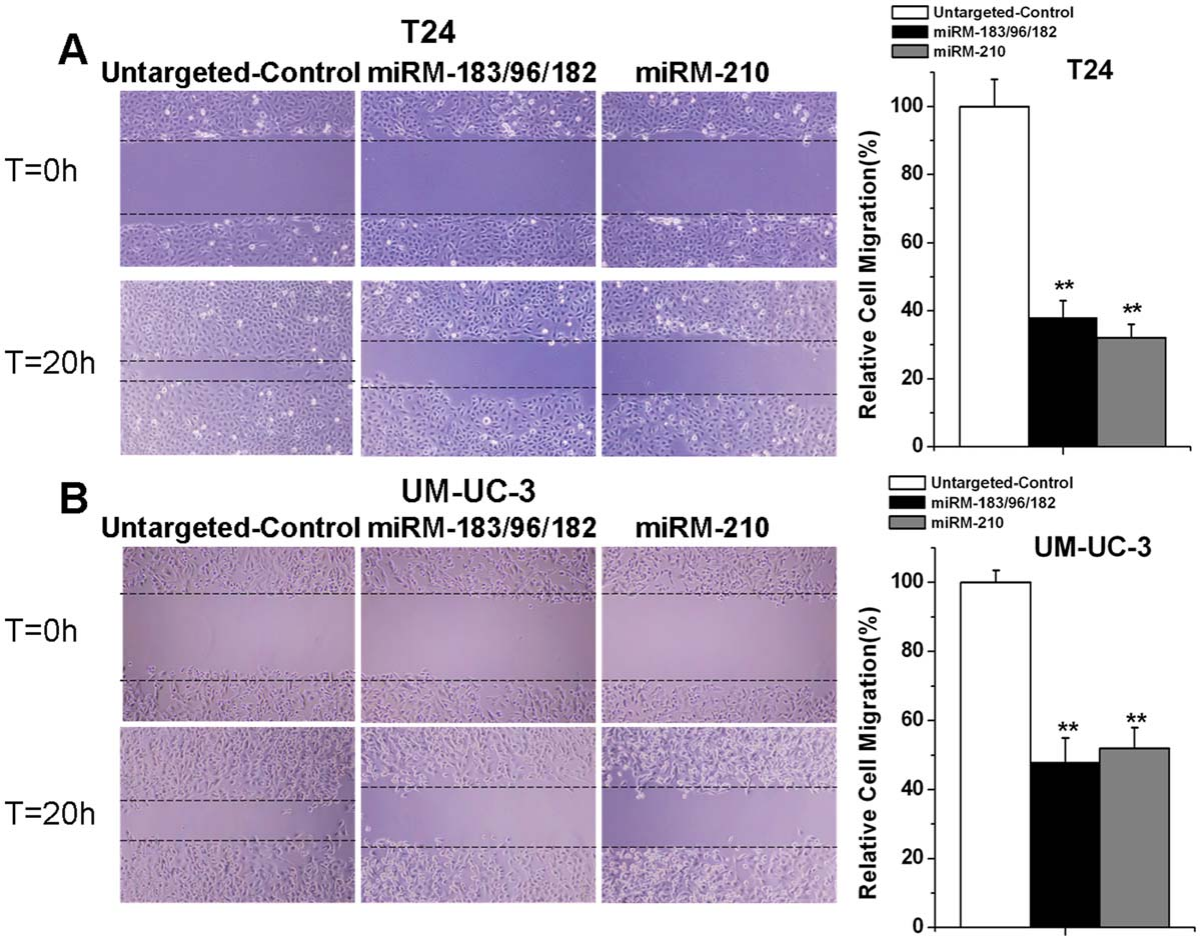
Effects of the synthetic miRNA-mowers on migration of bladder cancer cells. T24 and UM-UC-3 cells were transfected with the devices in 12-well plates. Cell migration was measured by the scratch assay. (A) miRM-183/96/182 and miRM-210 inhibited cell migration in T24 cells independently. (B) miRM-183/96/182 and miRM-210 inhibited cell migration in UM-UC-3 cells independently. Each experiment was performed at least three times and a representative picture was shown. Results were shown in mean ± SD. ** P<0.01, compared with the untargeted-control.

Cells were seeded in 12-well plates and transfected with the devices. Then we examined the role of each device on tumor cell migration by performing scratch assays. As shown in Fig. 4, the migration distances of cells in the groups transfected with the miRNA-mowers were significantly lower than those in the groups transfected with the untargeted-control.

## Discussion

It is largely recognized that miRNAs induce mRNA degradation via sequence-specific interactions. However, recent work has proposed that the major function of some miRNA-mRNA interactions is to block the miRNA, as opposed to suppress expression of the target genes [12]. Several experimental results support this new conception of miRNA regulation. In mammalian cells, the *PTENP1* pseudogene with conserved miRNA binding sites in its 3′UTR was shown to regulate *PTEN* expression through base-pairing interaction with the miRNAs. A similar phenomenon was also discovered in *KRAS* and its pseudogene *KRAS1P* [16]–[17]. Sequence-specific miRNA degradation was recently observed in marmoset T cells transformed with *Herpesvirus saimiri* (HVS). The virus encodes a small non-coding RNA–called HSUR1–that contains sites with extensive complementarity to miR-27a. Base pairing between miR-27a and HSUR1 can down-regulate the expression levels of miR-27a [18]. Based on these findings, we speculate that a natural small RNA molecule can be acting as a mower for miRNAs that bind to the specific recognition sites in its nucleotide sequence. So it seems reasonable that artificial miRNA regulatory devices with sequences partially complementary to miRNA of interest also have the ability to inhibit the corresponding endogenous miRNA activity and/or expression.

To test this hypothesis, we constructed synthetic devices containing multiple bulged miRNA binding sites and named them “miRNA-mowers”. Obviously the reason for choosing this name for the synthetic device is that it can “mow down” miRNA expression just like a lawn mower. The devices were designed to be modular, where the tandem binding sites could be changed by the others. Their expression was driven to high level by the strong SV-40 viral promoter in human bladder cancer cells. Using two validation experiments, we showed that the miRNA-mowers constructed by us were functional. Firstly, luciferase assays confirmed that activities of the luciferase reporters in the miRNA-mowers were suppressed in the two bladder cancer cells. Secondly, Real-Time qPCR was performed to detect the expression levels of target miRNAs in bladder cancer cells transfected with the devices, and we demonstrated that the amounts of these miRNAs were reduced by the corresponding miRNA-mower. According to these two findings, we conclude that the miRNA-mowers can functionally inhibit a single target miRNA or block a whole cluster of related miRNAs.

More and more studies have shown that miRNAs are frequently deregulated in many types of human cancers. As the critical roles of miRNAs in cancers are gradually explored, their applications as potential therapeutic targets have generated great interest in developing novel strategy for treating cancer [21]–[22]. Either individually or as a cluster, the expression levels of miR-96, miR-182, and miR-183 have been shown to be up-regulated in several cancers, including prostate cancer, breast cancer, lung cancer, medulloblastoma, bladder cancer and etc. [23]–[27]. Lin et al. found that miR-96 induced the proliferation and anchorage-independent growth of breast cancer cell lines [28]. Segura et al. found that miR-182 over-expression promoted the migration of human melanoma cells in vitro and their metastases in vivo [29]. Sarver et al. found that miR-183 functioned as an oncogene by increasing cancer cells migration [30]. miR-210 has emerged as a novel tumor biomarker regulated by hypoxia. The unique seed region of miR-210 distinguishes it from all of the other miRNAs. Expression of miR-210 was linked to tumor growth, invasion and poor patient survival [31]. Yang et al. discovered that miR-210 downregulation inhibited cell growth and induced cell apoptosis in human hepatoma cells [32]. Fasanaro et al. discovered that anti-miR-210 transfection decreased cell migration, inhibited cell growth and induced apoptosis [33]. These studies provide evidences for the important roles of the four selective miRNAs in the pathogenesis of human cancers. However, their effects on the phenotypes of bladder cancer cells remain largely unknown.

In this study, we have shown that miR-183-96-182 cluster or miR-210 suppression by the corresponding miRNA-mower not only inhibited growth and migration but also induced apoptosis in human bladder cancer cells. The inhibition of cell growth was due to the induction of cell apoptosis. These results suggested that miR-183/96/182 cluster and miR-210 play important roles in the regulation of biological behaviors of bladder cancer cells.

All the observed changes in phenotype should be mediated by miRNA-regulated genes. It has been demonstrated in the previous work that knockdown of the miR-183/96/182 cluster resulted in enrichment of apoptosis pathways and dysregulation of the PI3K/AKT/mTOR pathway [26]. Components of the PI3K/AKT/mTOR pathway were reported to be critical for tumorigenesis, including cellular proliferation, growth, survival and mobility [34]. There was also report demonstrating that this cluster inhibited zinc uptake via suppression of zinc transporters [23]. Zinc is an inhibitor of HIF-1α in human cancers that represses important genes involved in tumor progression, such as VEGF, MDR1 and Bcl2 [35]. miR-210 has been known to regulate hypoxia, which is important for cancer cell survival and invasion. Several miR-210 targets that influence cell growth, apoptosis, and migration have already been identified, such as E2F transcription factor 3 (E2F3) [36], fibroblast growth factor receptor like 1 (FGFRL1) [37], homeobox A1 (HOXA1) [38], FLICE-associated huge protein (FLASH)/caspase-8-associated protein-2 (Casp8ap2) [39] and Ephrin-A3 (EFNA3) [33]. Further analyses are needed to investigate their genetic regulation in the bladder cancer cells.

It should also be noted that the two miRNA-mowers are not absolutely efficient, because they have only resulted in marginal phenotypic changes in bladder cancer cells. There are several factors that may affect the efficiency of the devices. The function of a miRNA-mower depends not just on the number of miRNA binding sites, but also on the amount of mower RNAs relative to the amount of the endogenous miRNAs. To provide a more effective cancer treatment, several points may be taken into consideration. Addition of more miRNA binding sites to the devices can increase the concentration of the partially complementary sequences and will therefore increase the inhibitory effects of the miRNA-mowers. To express the devices at a high level, one can select more potential strong promoters for driving transcription and use viral vectors for delivering the miRNA-mowers. Some other possibilities remain to be shown.

In summary, anti-growth effects mediated by apoptosis and anti-migration effects were both induced by the synthetic miRNA-mowers targeting miR-183-96-182 cluster or miR-210 in the bladder cancer cells. Further researches are still needed to prove the utility of our synthetic biology platform in miRNAs functions studies and cancer treatments.

## Materials and Methods

### Cell lines and cell culture

Human bladder transitional cell carcinoma cell lines T24 and UM-UC-3 were purchased from the American Type Culture Collection (ATCC, Manassas, VA, USA). Both of them were maintained in RPMI-1640 (1640) media supplemented with 10% fetal bovine serum and 1% antibiotics (100 U/ml penicillin and 100 μg/ml streptomycin sulfates). Cells were routinely grown at 37°C in an atmosphere of 5% CO_2_.

### Creation of the miRNA-mowers

Bulged miRNA binding sites for the miR-96, miR-182, miR-183, and miR-210 and the linkers between them were designed and chemically synthesized. Either the combination DNA part for the miR-183-96-182 cluster containing 2 copies of binding sites for each cluster miRNA or the specific DNA part for miR-210 containing 6 copies of binding sites for miR-210 was cloned into siCHECKTM-2 luciferase vector (Promega, Madison, WI, USA) digested with *Xhol* and *Notl*. As an untargeted-control, a device with 6 tandem repeated binding sites complementary to no known miRNAs was also constructed by using the same methods as described above. Thorough descriptions of the related sequences were presented in Table S1 and Table S2.

### Cell transfection

Cells were plated twenty hours prior to transfection to achieve 70–80% confluency at the time of transfection. 1ug of each device was transfected into cells using Hiperfect Transfection Reagent (Invitrogen, Carlsbad, CA, USA) according to the manufacturer's protocols.

### Luciferase reporter assay

Cells were seeded in six-well plates (5×10^5^/well) and transfected with the miRNA-mowers or the untargeted-control. Luciferase activity was detected using the dual luciferase assay system (Promega, Madison, WI, USA) according to the manufacturer's instructions at 48 hours after transfection. The Renilla luciferase activity was normalized to the firefly luciferase activity. The inhibition (%) of luciferase expression was calculated by the following formula: Inhibition (%)  =  (relative luciferase activity in the untargeted-control − relative luciferase activity in the miRNA-mower)/relative luciferase activity in the untargeted-control ×100%. The experiments were performed in duplicate and repeated at least three times.

### RNA extraction and Real-Time qPCR

Total RNAs were isolated from T24 cells and UM-UC-3 cells using the TRIzol reagent (Invitrogen, Carlsbad, CA, USA) according to the manufacturer′s instructions. cDNAs were synthesized using the M-MLV Reverse Transcriptase (Promega, Madison, WI, USA). Real time PCR was carried out using the All-in-One^TM^ miRNA qRT-PCR Detection Kit (GeneCopoiea Inc, Rockville, MD, USA). U6 small nuclear RNA (snRNA) was selected as the endogenous control. The miRNA qPCR Primers were ordered from GeneCopoeia, Inc. The catalog numbers of All-in-One™ miRNA qPCR Primers were as follows: hsa-miR-96∼HmiRQP0852; hsa-miR-182∼HmiRQP0239; hsa-miR-183∼HmiRQP0244; has-miR-210∼HmiRQP0317; snRNA U6 ∼ HmiRQP9001 (The company did not provide sequence information of these Primers). The PCR mixtures were prepared according to the manufacturer's protocols with PCR conditions of 40 cycles of 15 sec at 95°C, 20 sec at 55°C, and 30 sec at 70°C on a ABI PRISM 7000 Fluorescent Quantitative PCR System (Applied Biosystems, Foster City, CA, USA). The relative expression of each miRNA was calculated using 2^−ΔΔCt^ methods. The inhibition (%) of miRNA expression was calculated by the following formula: Inhibition (%)  =  (relative miRNA expression level in cells transfected with the untargeted-control − relative miRNA expression level in cells transfected with the miRNA-mower)/relative miRNA expression level in cells transfected with the untargeted-control ×100%.

### Cell growth assay

The effects of the designed devices on cell proliferation were examined by MTT assay according to the reported methods [40]. 24, 48 or 72 hours post-transfection, 20 μl of MTT (5 mg/ml) was added to each well of a 96-well plate and the cells were cultured for 5 hours. Then the MTT medium mixtures were discarded and 100 μl of dimethyl sulfoxide (DMSO) was added to each well. Absorbance was measured at a wavelength of 490 nm (with 630 nm as the reference wavelength) using an ELISA microplate reader (Bio-Rad, Hercules, CA, USA). Assays were repeated at least three times.

### Cell apoptosis assay

Cell apoptosis was detected using an annexin V-fluorescein isothiocyanate (FITC)/propidium iodide (PI) apoptosis detection kit (BD, San Jose, CA, USA) according to the supplier's protocols. 48 hours post-transfection, cells were collected, centrifuged, and resuspended in 500 μl of 1×binding buffer. Annexin V-FITC (5 μl) and 10 μl PI were then added to each tube. The tubes were incubated in the dark at room temperature for 15 min. Cell apoptosis assay was performed immediately on a flow cytometry (EPICS, XL-4, Beckman, CA, USA). Each experiment was done at least three times.

### Cell migration assay

Cell motility was examined by scratch assay according to the methods previously described [41]. Cells were seeded in 12-well plates at a density of 1.0×10^5^ cells per well. We transfected cells with the devices and incubated the dishes at 37°C until cells reached 100% confluence. An artificial gap was then generated by scratching with a pipette tip. Photographic images were taken from each well immediately and again after 20 h using a digital camera system. The software program HMIAS-2000 was used to calculate the cell migration distance (μm). Each experiment was repeated at least three times.

### Statistical analyses

Data from all the experiments were expressed as mean ± the standard deviation (SD). Statistical significance was determined using Student's t-test or ANOVA and P <0.05 was considered statistically significant. All statistical tests were conducted by using SPSS version 17.0 software (SPSS, Chicago, IL, USA).

## Supporting Information

Table S1 MiRNA Sequences in the Study.
(DOC)

Table S2 Synthetic miRNA-Mower Sequences in the Vector.
(DOC)

Table S3 Delt-Ct values of Real-Time qPCR in both bladder cancer cells lines transfected with the synthetic devices.
(DOC)

## References

[1] Montironi R, Lopez-Beltran A (2005) The 2004 WHO classification of bladder tumors: a summary and commentary. Int J Surg Pathol 13(2): 143–153. 15864376 10.1177/106689690501300203

[2] Parekh DJ, Bochner BH, Dalbagni G (2006) Superficial and muscle-invasive bladder cancer: principles of management for outcomes assessments. J Clin Oncol 24(35): 5519–5527. 17158537 10.1200/JCO.2006.08.5431

[3] Lei AQ, Cheng L, Pan CX (2011) Current treatment of metastatic bladder cancer and future directions. Expert Rev Anticancer Ther 11(12): 1851–1862. 22117153 10.1586/era.11.181

[4] Racioppi M, D'Agostino D, Totaro A, Pinto F, Sacco E, et al (2012) Value of current chemotherapy and surgery in advanced and metastatic bladder cancer. Urol Int 88(3): 249–258. 22354060 10.1159/000335556

[5] Marta GN, Hanna SA, Gadia R, Correa SF, Silva JL, et al (2012) The role of radiotherapy in urinary bladder cancer: current status. Int Braz J Urol 38(2): 144–153. 22555038 10.1590/s1677-55382012000200002

[6] Carradori S, Cristini C, Secci D, Gulia C, Gentile V, et al (2012) Current and emerging strategies in bladder cancer. Anticancer Agents Med Chem 12(6): 589–603. 22043990 10.2174/187152012800617768

[7] Culler SJ, Hoff KG, Smolke CD (2010) Reprogramming cellular behavior with RNA controllers responsive to endogenous proteins. Science 330(6008): 1251–1255. 21109673 10.1126/science.1192128 PMC3171693

[8] Karlsson M, Weber W (2012) Therapeutic synthetic gene networks. Curr Opin Biotechnol 23: 1–9. 22305476 10.1016/j.copbio.2012.01.003

[9] Shankar S, Pillai MR (2011) Translating cancer research by synthetic biology. Mol Biosyst 7(6): 1802–1810. 21437339 10.1039/c1mb05016h

[10] Nissim L, Bar-Ziv RH (2010) A tunable dual-promoter integrator for targeting of cancer cells. Mol Syst Biol 6: 444. 21179016 10.1038/msb.2010.99 PMC3018173

[11] Xie Z, Wroblewska L, Prochazka L, Weiss R, Benenson Y (2011) Multi-input RNAi-based logic circuit for identification of specific cancer cells. Science 333(6047): 1307–1311. 21885784 10.1126/science.1205527

[12] Pasquinelli AE (2011) MicroRNAs and their targets: recognition, regulation and an emerging reciprocal relationship. Nat Rev Genet 13(4): 271–282. 10.1038/nrg3162 22411466

[13] Catto JW, Alcaraz A, Bjartell AS, De Vere White R, Evans CP, et al (2011) MicroRNA in prostate, bladder, and kidney cancer: a systematic review. Eur Urol 59(5): 671–681. 21296484 10.1016/j.eururo.2011.01.044

[14] Iorio MV, Croce CM (2012) microRNA involvement in human cancer. Carcinogenesis 33(6): 1126–1133. 22491715 10.1093/carcin/bgs140 PMC3514864

[15] Han Y, Chen J, Zhao X, Liang C, Wang Y, et al (2011) MicroRNA expression signatures of bladder cancer revealed by deep sequencing. PLoS One 6(3): e18286. 21464941 10.1371/journal.pone.0018286 PMC3065473

[16] Poliseno L, Salmena L, Zhang J, Carver B, Haveman WJ, et al (2010) A coding-independent function of gene and pseudogene mRNAs regulates tumour biology. Nature 465(7301): 1033–1038. 20577206 10.1038/nature09144 PMC3206313

[17] Muro EM, Mah N, Andrade-Navarro MA (2011) Functional evidence of post-transcriptional regulation by pseudogenes. Biochimie 93(11): 1916–1921. 21816204 10.1016/j.biochi.2011.07.024

[18] Cazalla D, Yario T, Steitz JA (2010) Down-regulation of a host microRNA by a Herpesvirus saimiri noncoding RNA. Science 328(5985): 1563–1566. 20558719 10.1126/science.1187197 PMC3075239

[19] Liu J, Carmell MA, Rivas FV, Marsden CG, Thomson JM, et al (2004) Argonaute2 is the catalytic engine of mammalian RNAi. Science 305(5689): 1437–1441. 15284456 10.1126/science.1102513

[20] Saetrom P, Heale BS, Snøve O Jr, Aagaard L, Alluin J, et al (2007) Distance constraints between microRNA target sites dictate efficacy and cooperativity. Nucleic Acids Res 35(7): 2333–2342. 17389647 10.1093/nar/gkm133 PMC1874663

[21] Cho WC (2012) MicroRNAs as therapeutic targets and their potential applications in cancer therapy. Expert Opin Ther Targets 16(8): 747–759. 22690697 10.1517/14728222.2012.696102

[22] Cho WC (2012) Exploiting the therapeutic potential of microRNAs in human cancer. Expert Opin Ther Targets 16(4): 345–350. 22339340 10.1517/14728222.2012.663354

[23] Mihelich BL, Khramtsova EA, Arva N, Vaishnav A, Johnson DN, et al (2011) miR-183-96-182 cluster is over-expressed in prostate tissue and regulates zinc homeo-stasis in prostate cells. J Biol Chem 286(52): 44503–44511. 22045813 10.1074/jbc.M111.262915 PMC3247959

[24] Lehmann U, Streichert T, Otto B, Albat C, Hasemeier B, et al (2010) Identification of differentially expressed microRNAs in human male breast cancer. BMC Cancer 10: 109. 20331864 10.1186/1471-2407-10-109 PMC2850898

[25] Zhu W, Liu X, He J, Chen D, Hunag Y, et al (2011) Overexpression of members of the microRNA-183 family is a risk factor for lung cancer: a case control study. BMC Cancer 11: 393. 21920043 10.1186/1471-2407-11-393 PMC3183044

[26] Weeraratne SD, Amani V, Teider N, Pierre-Francois J, Winter D, et al (2012) Pleiotropic effects of miR-183∼96∼182 converge to regulate cell survival, proliferation and migration in medulloblastoma. Acta Neuropathol 123(4): 539–552. 22402744 10.1007/s00401-012-0969-5 PMC6172007

[27] Yamada Y, Enokida H, Kojima S, Kawakami K, Chiyomaru T, et al (2011) MiR-96 and miR-183 detection in urine serve as potential tumor markers of urothelial carcinoma: correlation with stage and grade, and comparison with urinary cytology. Cancer Sci 102(3): 522–529. 21166959 10.1111/j.1349-7006.2010.01816.x

[28] Lin H, Dai T, Xiong H, Zhao X, Chen X, et al (2010) Unregulated miR-96 induces cell proliferation in human breast cancer by downregulating transcriptional factor FOXO3a. PLoS One 5(12): e15797. 21203424 10.1371/journal.pone.0015797 PMC3009749

[29] Segura MF, Hanniford D, Menendez S, Reavie L, Zou X, et al (2009) Aberrant miR-182 expression promotes melanoma metastasis by repressing FOXO3 and microphthalmia-associated transcription factor. Proc Natl Acad Sci U S A 106(6): 1814–1819. 19188590 10.1073/pnas.0808263106 PMC2634798

[30] Sarver AL, Li L, Subramanian S (2010) MicroRNA miR-183 functions as an oncogene by targeting the transcription factor EGR1 and promoting tumor cell migration. Cancer Res. 70(23): 9570–9580. 10.1158/0008-5472.CAN-10-2074 21118966

[31] Camps C, Buffa FM, Colella S, Moore J, Sotiriou C, et al (2008) hsa-miR-210 is induced by hypoxia and is an independent prognostic factor in breast cancer. Clin Cancer Res 14(5): 1340–1348. 18316553 10.1158/1078-0432.CCR-07-1755

[32] Yang W, Sun T, Cao J, Liu F, Tian Y, et al (2012) Downregulation of miR-210 expression inhibits proliferation, induces apoptosis and enhances radiosensitivity in hypoxic human hepatoma cells in vitro. Exp Cell Res 318(8): 944–954. 22387901 10.1016/j.yexcr.2012.02.010

[33] Fasanaro P, D'Alessandra Y, Di Stefano V, Melchionna R, Romani S, et al (2008) MicroRNA-210 modulates endothelial cell response to hypoxia and inhibits the receptor tyrosine kinase ligand Ephrin-A3. J Biol Chem 283(23): 15878–15883. 18417479 10.1074/jbc.M800731200 PMC3259646

[34] Wu P, Hu YZ (2010) PI3K/Akt/mTOR pathway inhibitors in cancer: a perspective on clinical progress. Curr Med Chem 17(35): 4326–4341. 20939811 10.2174/092986710793361234

[35] Nardinocchi L, Pantisano V, Puca R, Porru M, Aiello A, et al (2010) Zinc downregulates HIF-1α and inhibits its activity in tumor cells in vitro and in vivo. PLoS One 5(12): e15048. 21179202 10.1371/journal.pone.0015048 PMC3001454

[36] Giannakakis A, Sandaltzopoulos R, Greshock J, Liang S, Huang J, et al (2008) miR-210 links hypoxia with cell cycle regulation and is deleted in human epithelial ovarian cancer. Cancer Biol Ther 7(2): 255–264. 18059191 10.4161/cbt.7.2.5297 PMC3233968

[37] Huang X, Ding L, Bennewith KL, Tong RT, Welford SM, et al (2009) Hypoxia-inducible mir-210 regulates normoxic gene expression involved in tumor initiation. Mol Cell. 35(6): 856–967. 10.1016/j.molcel.2009.09.006 PMC2782615 19782034

[38] Noman MZ, Buart S, Romero P, Ketari S, Janji B, et al (2012) Hypoxia-inducible miR-210 regulates the susceptibility of tumor Cells to lysis by cytotoxic T cells. Cancer Res. 72(18): 4629–4641. 10.1158/0008-5472.CAN-12-1383 22962263

[39] Kim HW, Haider HK, Jiang S, Ashraf M (2009) Ischemic preconditioning augments survival of stem cells via miR-210 expression by targeting caspase-8-associated protein 2. J Biol Chem 284(48): 33161–33168. 19721136 10.1074/jbc.M109.020925 PMC2785158

[40] MosmannT (1983) Rapid colorimetric assay for cellular growth and survival: application to proliferation and cytotoxicity assays. J Immunol Methods 65(1–2): 55–63. 6606682 10.1016/0022-1759(83)90303-4

[41] Liang CC, Park AY, Guan JL (2007) In vitro scratch assay: a convenient and inexpensive method for analysis of cell migration in vitro. Nat Protoc 2(2): 329–333. 17406593 10.1038/nprot.2007.30

