# Supplementary material for: Synthetic miRNA-Mowers Targeting miR-183-96-182 Cluster or miR-210 Inhibit Growth and Migration and Induce Apoptosis in Bladder Cancer Cells
Source: PLoS One. 2012 Dec 17;7(12):e52280. doi: 10.1371/journal.pone.0052280 (PMC3524115; doi:10.1371/journal.pone.0052280)
Supplement: Table S1 — MiRNA Sequences in the Study. (DOC) [file pone.0052280.s001.doc]

**Supplementary Table1. miRNA Sequences in the Study**

| miRBase Accession | miRNA Name | miRNA Sequence (5’→3’) |
| --- | --- | --- |
| MIMAT0000267 | miR-210 | CUGUGCGUGUGACAGCGGCUG A |
| MIMAT0000259 | miR-182 | UUUGGCAAUGGUAGAACUCACACU |
| MIMAT0000261 | miR-183 | UAUGGCACUGGUAGAAUUCACU |
| MIMAT0000095 | miR-96 | UUUGGCACUAGCACAUUUUUGCU |

Note: The underlined parts are seed sequences (miRNA nucleotides 2–8).
