# Supplementary material for: Synthetic miRNA-Mowers Targeting miR-183-96-182 Cluster or miR-210 Inhibit Growth and Migration and Induce Apoptosis in Bladder Cancer Cells
Source: PLoS One. 2012 Dec 17;7(12):e52280. doi: 10.1371/journal.pone.0052280 (PMC3524115; doi:10.1371/journal.pone.0052280)
Supplement: Table S2 — Synthetic miRNA-Mower Sequences in the Vector. (DOC) [file pone.0052280.s002.doc]

**Supplementary Table2. Synthetic miRNA Mower Sequences in the Vector**

| miRNA Mower Name | miRNA Mower Sequence |
| --- | --- |
| miRM-183/96/182 | *CTCGAG*  AGTGTGAGTTCTGAAGTTGCCAAACTTC  AGTGAATTCTCAACGTGCCATACTTC  AGCAAAAATGTTAGCGTGCCAAACTTC  AGTGTGAGTTCTGAAGTTGCCAAACTTC  AGTGAATTCTCAACGTGCCATACTTC  AGCAAAAATGTTAGCGTGCCAAA  *GCGGCCGC* |
| miRM-210 | *CTCGAG*  TCAGCCGCTGGTGAACGCACAGCTTC  TCAGCCGCTGGTGAACGCACAGCTTC  TCAGCCGCTGGTGAACGCACAGCTTC  TCAGCCGCTGGTGAACGCACAGCTTC  TCAGCCGCTGGTGAACGCACAGCTTC  TCAGCCGCTGGTGAACGCACAGCTTC  *GCGGCCGC* |
| miRM-untargeted-control | *CTCGAG*  CCCGG  AAGTTTTCAGAAAGCTAACA  AAGTTTTCAGAAAGCTAACA  AAGTTTTCAGAAAGCTAACA  AAGTTTTCAGAAAGCTAACA  AAGTTTTCAGAAAGCTAACA  AAGTTTTCAGAAAGCTAACA  *GCGGCCGC* |

Note: miRM represents miRNA Mower; *CTCGAG*, restriction enzyme recognition sequence for *Xhol; GCGGCCGC,* restriction enzyme recognition sequence for *Notl;* CTTC, linker; CCCGG, linker; Shadowed parts are bulged sites that are mispaired opposite miRNA positions 9–12. AAGTTTTCAGAAAGCTAACA : an untargeted control sequence not complementary to any known microRNAs described by Ebert MS, et al [1].

**Reference**

[1]. Ebert MS, Neilson JR, Sharp PA. MicroRNA sponges: competitive inhibitors of small RNAs in mammalian cells. Nat Methods. 2007(9):721-6.
