# Supplementary material for: Synthetic miRNA-Mowers Targeting miR-183-96-182 Cluster or miR-210 Inhibit Growth and Migration and Induce Apoptosis in Bladder Cancer Cells
Source: PLoS One. 2012 Dec 17;7(12):e52280. doi: 10.1371/journal.pone.0052280 (PMC3524115; doi:10.1371/journal.pone.0052280)
Supplement: Table S3 — Delt-Ct values of Real-Time qPCR in both bladder cancer cells lines transfected with the synthetic devices. (DOC) [file pone.0052280.s003.doc]

**Supplementary Table 3. Delt-Ct values of Real-Time qPCR in both bladder cancer cell lines transfected with the synthetic devices**

| miRNAs | miRM-183/96/182 | | miRM-210 | | untargeted-control | |
| --- | --- | --- | --- | --- | --- | --- |
|  | ΔCt/T24 | ΔCt/UM-UC-3 | ΔCt/T24 | ΔCt/UM-UC-3 | ΔCt/T24 | ΔCt/UM-UC-3 |
| miR-96 | 4.54 | 4.15 | 3.65 | 3.29 | 3.53 | 3.23 |
| miR-182 | 4.19 | 4.10 | 3.17 | 3.25 | 3.28 | 3.15 |
| miR-183 | 4.55 | 4.43 | 3.52 | 3.15 | 3.39 | 3.21 |
| miR-210 | 1.04 | 0.92 | 2.88 | 2.65 | 1.12 | 0.86 |
| Note: The results were obtained from one of three independent experiments. The median in each triplicate was used to calculate relative miRNA concentrations .ΔCt = Ct _medianmiRNA_ − Ct _mediansnRNAU6_. | | | | | | |
